# Collaborating neuroscience online: The case of the Human Brain Project forum

**DOI:** 10.1371/journal.pone.0278402

**Published:** 2022-12-07

**Authors:** Ann-Christin Kreyer, Lucy Xiaolu Wang

**Affiliations:** 1 Max Planck Institute for Innovation and Competition, München, Germany; 2 Munich Graduate School of Economics, Ludwig-Maximilians-University Munich, München, Germany; 3 Department of Resource Economics, University of Massachusetts, Amherst, Massachusetts, United States of America; 4 Canadian Centre for Health Economics, University of Toronto, Toronto, Ontario, Canada; Sabanci University: Sabanci Universitesi, TURKEY

## Abstract

This paper analyzes user interactions on the public-access online forum of the Human Brain Project (HBP), a major European Union-funded neuroscience research initiative, to understand the utility of the Forum for collaborative problem solving. We construct novel data using discussion forum posts and detailed user profiles on the HBP Forum. We find that HBP Forum utilization is comparable to that of a leading general-interest coding platform, and that online usage metrics quickly recovered after an initial Covid-19-related dip. Regression results show that user interactions on the Forum are more active for questions on programming and in HBP core areas. Further, Cox proportional hazard analyses show that such problems are solved faster. Forum posts with users from different countries tend to be discussed more actively but solved slower. Higher shares of administrator support tend to solve problems faster. There are no clear patterns regarding gender and seniority. Our results suggest that building novel collaborative forums can support researchers working on complex topics in challenging times.

## 1. Introduction

Neurological disorders are the leading cause of disability and the second leading cause of death worldwide, accounting for 9 million deaths (16.5% of total global deaths) and the loss of 276 million disability-adjusted life years in 2016 [1]. Most brain diseases have no cure, and many existing treatments are very expensive. Meanwhile, there is growing public and private investment in artificial intelligence (AI) for health care projects, with health care being the most invested sector by AI investors [2]. Although many life science areas, including neuroscience, are traditionally more laboratory- and experimentally-based, both Covid-19 and the massive global cost of neurological diseases heighten the need to harness digitization in upstream health care markets. Since 2013, there have been different brain science initiatives launched in Europe, US, Israel, Japan, and China [3] as well as an emerging international brain initiative [4], leading to a burgeoning “brain race” [5].

This paper studies the public-access online forum of one of the earliest brain initiatives, the Human Brain Project (HBP). Launched in 10/2013, the HBP is a flagship science initiative in the European Commission’s Future and Emerging Technologies program and recipient of a 10-year, €1 billion grant. The HBP aims to advance brain research and improve treatments for neurological diseases by merging neuroscience with information and communication technology (ICT) including computational science, robotics, and artificial intelligence [6]. As of 2021, a total of 179 institutions from over 20 countries had participated in the HBP. As well as making grants to research partners, the HBP allocates resources to build digital platforms, including a public-access online HBP Forum. As a major public discussion channel of the HBP, the utilization and collaborative problem-solving on the HBP Forum offers a valuable case study of institutional design to facilitate scientific collaboration.

We construct a novel dataset to examine whether and to what extent the HBP Forum is actively used, what factors are tied with richer online user interactions, and whether the HBP Forum offers an effective platform for problem-solving. We collected data from public sources to capture all user interactions and discussion content on the HBP Forum as well as characteristics of forum user profiles (e.g., demographics, institutions, scientific areas). We categorize the discussion threads based on the nature of topics, and further identify whether, when, and by whom a question has been solved. Data reveal that the HBP Forum is well-utilized and remains resilient during Covid-19, reflected in both the extensive margin of usage and intensive margin of user interactions. With the novel data constructed, this paper offers the first systematic empirical analyses of the utilization and performance of the HBP Forum.

We employ regression analyses to investigate what factors are associated with richer user interactions measured by the numbers of user replies per post within a quarter. We analyze covariates related to the content of discussed topics, the technical aspects, and the demographic and institutional profile of users who post the initial questions and users who reply. We further create a content-based measure of whether and when each question raised is solved effectively. We define a post as solved effectively if the asking user confirms the proposed solution; when direct confirmation is not available, we label the solution status based on the content and co-users’ confirmation. We then utilize Cox proportional hazard models to analyze the time taken to solve a posted problem and covariates that accelerate problem-solving. We find that questions closely tied with HBP platforms and questions on programming issues with a higher share of explicit code in communications generate more discussions, especially when participating users are geographically more diverse. Questions posted on the Forum are solved faster when HBP administrators participate, and when code snippets are shared. Richness of interaction and likelihood of solution appear to be independent of participating users’ HBP affiliation status.

This paper contributes to two strands in the literature. First, our paper contributes to studies about knowledge-sharing platforms by studying a large-scale, multinational scientific forum. As research specialization increases, knowledge-sharing and collaboration become increasingly essential for knowledge creation [7, 8], which further promotes diversity in the process [9, 10]. Knowledge diffusion can be spurred by offline research institutions that further advance scientific discoveries [11, 12]. With the rise of remote work, online discussion forums spur knowledge sharing and creation with evolving communities and flat hierarchies [13–15]. Prior studies have examined patterns and drivers of organizational sharing in sub-national and proprietary platforms [16–19]. Recent qualitative studies suggest that life science platforms can function well by pooling resources from interdisciplinary areas [20, 21]. We further offer a quantitative study of an online life science forum backed by a supranational organization.

Second, studies on digitization in health care often focus on the adoption and utilization of health information technologies (HIT) among downstream users (e.g., health providers), and our study complements prior work by examining a research-oriented digital forum for upstream users (i.e., neuroscientists). Studies find HIT improve health outcomes mainly for complex conditions or specific populations [22–25], and that HIT can complement other programs in e.g. combating the opioid crisis [26]. However, the increases in costs arising from HIT are also substantial [27], although the cost burdens are less concerning in IT-intensive locations that provide complementary assets [28]. Given that brain-related diseases are mostly complex and effective treatments are rare, digitization can provide new channels to spur global research collaborations for treatments. Some studies hypothesize that digital platforms can help advance neurosciences [29, 30], and our paper provides the first systematic analysis on how a digital forum is used by neuroscientists.

In addition, our study has policy implications for the design of online institutions for life sciences research. Both novel design and effective utilization of online institutions have become increasingly important given the disruption arising from Covid-19, which is reflected in the creation of the National Virtual Biotechnology Laboratory (NVBL, science.osti.gov/nvbl) by the US Department of Energy (DOE) as a consortium of DOE national laboratories. The pre-Covid-19 experience of the HBP offers a setting to understand how to proactively build institutional capacity that remains resilient during a disruptive period such as a pandemic. While it is difficult to evaluate the long-term impact of such projects, our analyses of contemporary performance on the forum can help inform policy regarding certain aspects of online institutional designs.

## 2. Background and data construction

### 2.1 The Human Brain Project (HBP) and the HBP Forum

**Fig 1:**
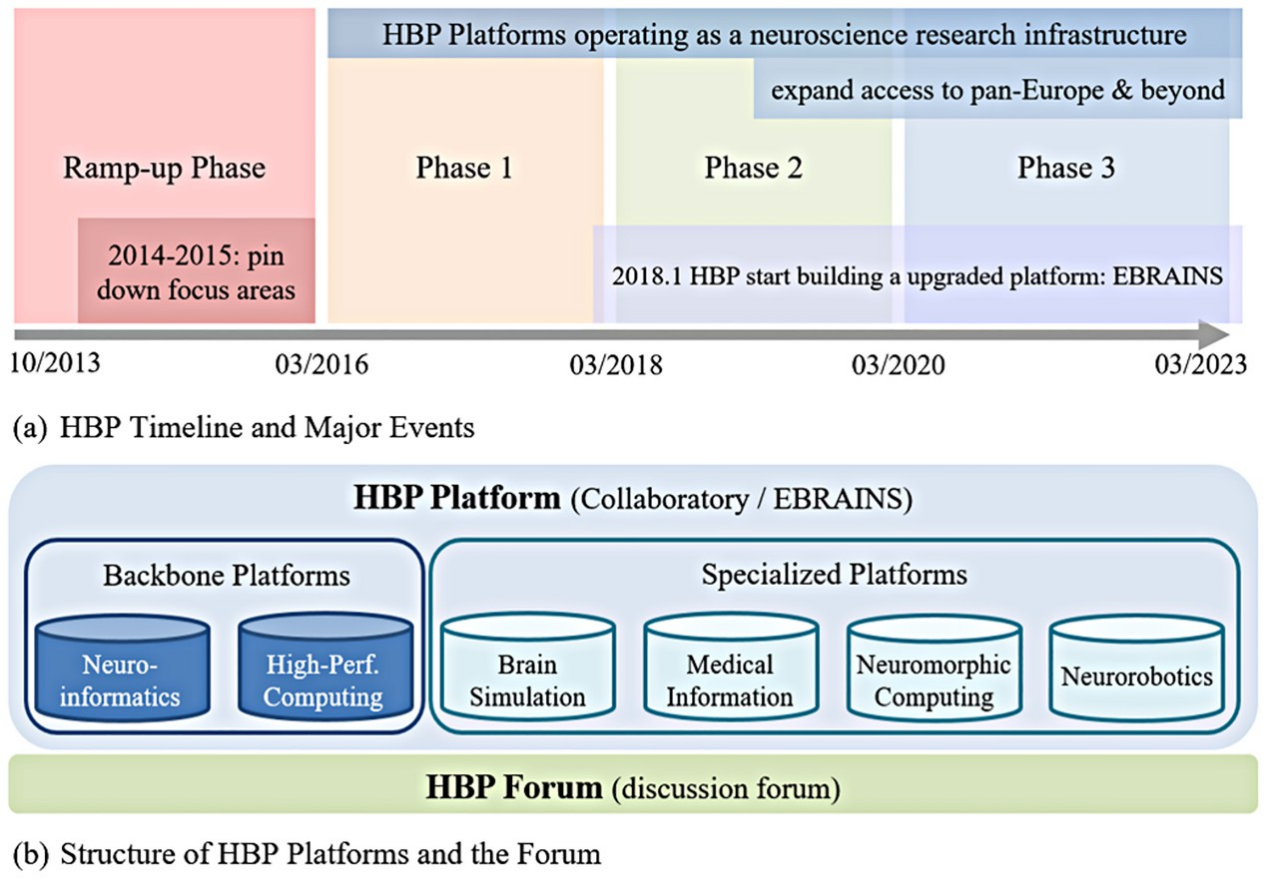
The organization of the HBP platforms and the HBP Forum. Notes: Author’s graph. Panel (a) depicts major milestones in the HBP timeline. Panel (b) provides an overview of the different AI-powered platforms built during the HBP and the broad relationship with the HBP Forum. While accessing certain areas within the HBP platforms requires additional authorization, the HBP Forum is publicly accessible and allows for decentralized knowledge sharing across the globe. Sources: https://www.humanbrainproject.eu/en/ and https://forum.humanbrainproject.eu/.

The HBP was launched in 2013, finished a ramp-up phase in 2016, and entered three special grant agreement phases in 2016, 2018, and 2020 (Fig 1(a)). The HBP includes 12 sub-project areas, including six generic topic areas (i.e., mouse brain organization, human brain organization, systems and cognitive neuroscience, theoretical neuroscience, management and coordination, ethics and society) and six platform-related sub-projects (i.e., neuroinformatics, brain simulation, high-performance analytics and computing, medical informatics, neuromorphic computing, and neurorobotics). Fig 1(b) shows the structure of the platforms. Access to the HBP platforms is granted to applicants from partner institutions; other users can request an account but are evaluated on a case-by-case basis. Besides general areas, research teams build project-specific private repositories, and such data are only accessible to related team members. From 01/2018, the HBP began building EBRAINS on the new EU neuroscience supercomputing centers. The sub-platforms maintain the same focus and can perform better. The HBP platforms are hosted on a centralized access point: on HBP Collaboratory from 03/2016-09/2021, transitioning thereafter to EBRAINS as the new host, which remains current to date.

The HBP Forum was launched in July 2015 as an integral connecting part of the HBP platform infrastructure. The Forum serves as a public discussion website about HBP-related topics, including questions on HBP-related activities in general, neuroscience progress, and programming challenges. Serving as the HBP’s “Stack Overflow”, topics raised and discussed in the Forum are public and can be read without registering an account, but only users with an account in the Forum can reply to or comment on topics raised in the Forum. Since the Forum is designed for public discussion, anyone interested in participating can create an account. Users do not have to be HBP-affiliated to use the Forum, and users with an HBP account can use the same account for the Forum. Therefore, the HBP Forum is designed to facilitate informal collaboration and knowledge-sharing between researchers within and beyond the HBP community.

In the absence of detailed project-level data, user interactions in the public HBP Forum are the best available source to analyze HBP platform utilization and real outcomes of the Forum for the neuroscience research community. In addition, the HBP Forum retained the same structure and functionality during the transition of the HBP platform host starting in 09/2021, making the Forum a consistent measure of user activity. There were 534 posts on the Forum during 07/2015-03/2021, with 2,492 total replies and 550,175 total views. The collection and analysis method complied with the terms and conditions for the source of the data.

### 2.2 Post-level HBP Forum data construction

We retrieved the full text of all posted threads available on the public HBP Forum between 07/2015 and 03/2021 (last accessed on 03/31/2021), and cross-checked the relationship database. We processed the rich text data and extracted information on the topics discussed in each post, the timestamp and content of each post and reply, and the number of total views for each post. We then merged in user-level data (see section 2.3) to the post-level data to capture the nuances on who interacts with whom, and how this differs by type of post. Based on the post-level data, we cleaned and organized the data at both the post-level including each complete thread of discussion following the initial question or topic posted and reply-level including each individual reply to a post. We have obtained approval and IRB exemption from the Data Protection Officer of the Max Planck Society and were confirmed that all data used are compliant with relevant sources and current regulation. During our sample period, a total of 534 posts were initiated by 208 of 283 total active users. We define active users are users who ever posted on the platforms, excluding registered users who never posted anything. On average, each post was viewed 1,030 times and received 3.7 replies. Across discussion topic categories, neurorobotics was the most popular, with 325 topics (60.8% of total) and 152 users (53.7% of total), followed by technical support (S1 Fig).

To capture nuances in HBP Forum discussions, we categorized each Forum post in two independent ways. First, we obtained the content-based sub-categories tagged by the Forum and grouped them into six major topic categories: neuromorphic, brain simulation/modeling, neurorobotics, technical support, organization, and others. Second, we analyzed all posts and manually categorized whether each post had a query to be solved, and if so, whether this query was solved by a HBP administrator/moderator or by users in the community. When multiple solutions were offered, we used the first solution timestamp to construct solving time. If an answer was provided first by a user and further clarified/confirmed by an administrator (2.5% of all posts), we classified it as administrator-solved. Posts that did not raise a question are classified as informational (i.e., were not question-oriented, and thus could not be solved). Two informational posts were re-categorized as questions, as users asked follow-up questions and were answered. Our results are robust to dropping or re-categorizing these two cases. Third, we recorded the timestamp when a query is solved. If an initial question was solved but inspired new questions and answers within the same post, we labelled such posts as multi-question posts and recorded information for each sub-question. In addition, we assigned two indicators for each post to capture if code snippets are included, and whether the topic being discussed is specifically related to HBP platforms (i.e., not a generic question).

To understand user diversity, equity, and inclusion on the platform, we further assigned indicators for whether a given post was created by a female user, whether it contained code snippets, whether the initial posting user was affiliated with an HBP partner institution, whether the initial post was created by a senior user, and the country where the user is based according to his/her main employer. Before aggregating the data to post- and post-quarter levels, we calculated the share of users who are female, share of replies that contain code, share of users who are more senior, and number of users affiliated with an HBP partner. In this way, we construct the dataset not only using the content of questions, but also to understand the type of questions being discussed and how diverse the Forum community is.

### 2.3 User-level data construction

We further constructed a user-level database to capture details about who interacts with whom, and why. We combined information from multiple sources, including the HBP Forum, HBP PLUS (i.e., a user profile database maintained by the HBP for statistical reporting purposes for which users can opt in), HBP websites profiling key team members across project areas, and HBP YouTube channels with archived information on past team members. Specifically, we obtained the list of users, online usernames, real names, and institutional affiliations whenever publicly available for active users registered in the HBP Forum database. We used multiple matching algorithms to merge the Forum data with other sources based on details including full name, institutional affiliation, country of residence, contact details, gender, fields of experience, and highest level of education. We matched the users with the information we had collected from HBP promotional videos on YouTube and the internal HBP user database, HBP PLUS. The matching was based on the usernames and the real names using STATA’s fuzzy matching algorithm “matchit” and the “merge” command. Each match was further verified manually. Where necessary, we supplemented these data with manual collection and disambiguation using Google Scholar searches, LinkedIn profiles, and institutional webpages. The dataset is anonymized and aggregated at the post-level.

### 2.4 Descriptive statistics

In each complete calendar year during our sample period, there are on average 98 posts and 366 replies posted in the Forum (Table 1, Panel A). Per quarter, on average 23 posts are raised with a total of 85 replies, equivalent to about 3.7 replies per post (excluding the initial post). Most of the replies (i.e., about 90%) come within three months, and it takes 16 days on average to solve a question raised (Table 1, Panel B). Both numbers suggest active usage of the Forum within a fairly short time window. Furthermore, there is a fair amount of heterogeneity in post-level interactions. Some posts are discussed and solved quickly, while others generate lively follow-up questions and discussions that offer more learning and collaboration opportunities.

**Table 1:** Summary statistics.

Dependent variables	N	Mean	Std. Dev.	min	max
***Panel A*: *Forum usage aggregate statistics***					
# posts per year (2016–2020)	5	98	54.09	56	190
# yearly replies (2016–2020)	5	366.2	219.02	178	717
# posts per year	7	76.29	57.67	21	190
# yearly replies	7	280.57	231.04	60	717
# post per quarter	23	23.22	14.4	6	57
# replies per quarter	23	85.39	62.54	16	231
# replies per quarter (≤ 6 months)	23	79.78	60.68	16	230
# replies per quarter (≤ 3 months)	23	77.04	58.03	13	215
# replies ≤ 3 months per posts	23	3.12	.9	1.85	4.67
***Panel B*: *Post-level***		Full Sample		Survival Sample	
		Freq.	%	Freq.	%
Total Posts		534	100	465	100
Information		69	12.92	0	0
Unsolved posts		84	15.73	84	18.06
Solved posts		381	71.35	381	81.94
Admin solved		312	58.43	312	67.10
User solved		69	12.92	69	14.84
Post w/ HBP platform relevance		456	85.39	443	95.27
Post w/o HBP platform relevance		78	14.61	22	4.73
Post w/ code		248	46.44	243	52.26
Post w/o code		286	53.56	220	47.74
Multi-thread posts		110	20.60	105	22.58
Average Solving Time		15.57 days		15.69 days	
***Panel C*: *User statistics***					
Users		260	100	233	100
Fully identified users		188	72.31	172	73.82
Non-admin users		211	81.15	188	80.69
Admin users		49	18.85	45	19.31
Male		211	86.48	194	88.18
Female		33	13.52	26	11.82
HBP affiliation		142	74.35	131	74.86
No HBP affiliation		49	25.65	44	25.14
User seniority		197	100	181	100
Undergraduate students		9	4.57	9	4.97
Graduate students		76	38.58	74	40.88
Junior researchers		28	14.21	24	13.26
Senior researchers		36	18.27	28	15.47
Senior software engineers		33	18.27	32	17.68
Non-academic		15	7.61	14	7.73

Notes: Panel (a) reports summary statistics of all discussion posts that appeared on the HBP Forum during the period 07/2015 and 03/2021. Panels (b) and (c) further disentangle characteristics for the full and focused samples at the post-level and user-level, respectively.

Out of the 534 total posts, 465 posts (87%) pose a question and the other 69 posts (13%) are informational posts (Table 1, Panel B). Informational posts address topics such as research diversity and explanations of different research tools. Of the 465 posts posing questions, 381 (82%) are solved, either by forum administrators (67%) or by users (15%). The share of unsolved posts on the Forum (18%) is smaller than that on Stack Overflow (29%) [31]. Considering all posts, most questions (85%) are directly related to the HBP platforms. Programming questions with code snippets account for 46% of all posts and 53% of question-oriented posts, including various levels of code intensity. Posts without code snippets often comprise organizational and application-related questions (S2–S4 Figs provide a few examples).

For the survival analyses, we focus on the initial question posted, excluding follow-up questions inspired within a given thread. We also exclude informational posts, as they pose no questions to be solved. These criteria result in our sample for the survival analyses with 465 observations. The survival sample has a similar distribution to the full sample regarding post-level and user-level characteristics, including share of posts with code, posts related to the HBP platforms, share of posted questions solved, and fully identified users (Table 1, Panel C).

There are 260 active users in our full sample, among which we fully identified 188 users (72%) regarding demographics, affiliation, and expertise. Unidentified users do not use their real names in their profile or in posts, and we do not have enough information to back up their identity. A large share of identified users is quite active, and most of them are affiliated with an HBP partner institution. At the user level (Table 1, Panel C), most active users of the HBP Forum are male (86%), slightly lower than the male rate on Stack Overflow during 2015–2020 (>90%). The share of female users (14%) is higher than that in Stack Overflow (i.e., 6% in Europe), but lower than the share of female neuroscientists worldwide (25%) (source: https://insights.stackoverflow.com/survey/2020) [32]. About 81% of Forum users are voluntary users (i.e., not administrators). 74% of users are affiliated with an HBP partner institution and are members (but not leaders) of HBP sub-projects. 92% of the active users are students or academic employees (e.g., researchers and software engineers). Non-academic users comprise computer scientists employed in private companies or self-employed entrepreneurs. Seniority level is coded for all active users. Nine users changed seniority level during our sample period and are coded accordingly. Most Forum users are graduate students (39%). Junior researchers (post-doctoral scholars and assistant professors) make up 14% of users, while senior researchers (associate and full professors) make up 18%. Finally, 18% of active users are senior software engineers employed by a research institution. We group senior researchers and senior software engineers together in our analyses.

Geographically, active users are based in 27 countries, with the top six countries accounting for about 76% of the users: Germany (30%), Switzerland (21%), United Kingdom (8%), Italy (7%), United States (6%), and France (3%). On average, we observe users participated from 1.7 countries per post. The geographic diversity peaks in 2018 with 1.94 countries per post and is then stable throughout the observed window of time.

**Fig 2:**
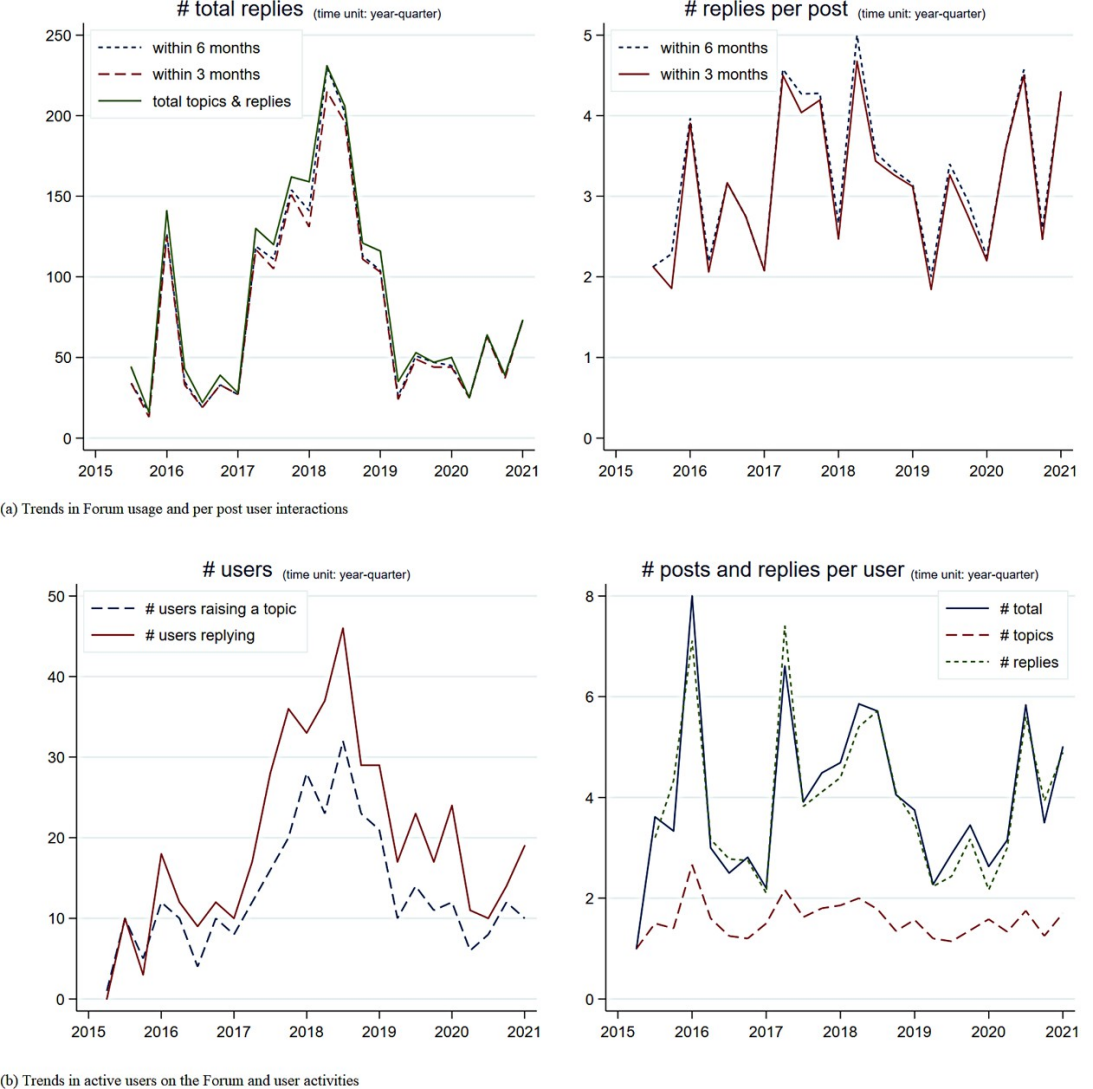
HBP Forum usage: Trends in posts, replies, and users. Note: Panel (a) depicts aggregate utilization of the Forum at a quarterly-level to the first quarter of 2021. Panel (b) offers a complementary view of the data from users’ perspective and shows aggregate user activity over time, both at the extensive margin (i.e., # active users) and at the intensive margin (i.e., # posts and replies per active user) per quarter.

Fig 2 depicts the trends in Forum usage, measured by aggregate post-level interactions, total active user engagement, and corresponding disaggregated numbers per post. Forum usage peaked in 2018 with the largest volume of post-level interactions and active users; this is consistent with a “deadline effect” coinciding with the conclusion of HBP Phase One, when users may have been finalizing results for end-of-phase reports. The numbers stabilize to around 50 new posts and 15 active users per quarter in 2019. The number of replies stabilizes to 3–4 per post, and each active user replies 4–5 times per quarter. Despite a drop in usage during 2020, user interaction rose substantially in late 2020 and early 2021.

## 3. Empirical analyses and results

### 3.1 What drives richer online user interactions?

To examine the factors that drive richer online user interactions, we aggregated the data to the post-quarter level. The data structure is not a panel as we often have only one observation over time per post. The data set contains 599 observations of 534 posts, where 58 posts containing replies from more than a quarter (i.e., 3 months) later contribute to the number of observations exceeding posts. We perform regression analysis at the post-quarter level using the following equation:

$$y_{it}=\delta_{t}+\eta HBP_{platform_{i}}+\gamma X_{it}+\epsilon_{it}$$ (1)

Here *y*_*it*_, is the number of replies for post *i* in quarter *t*. $HBP_{platform_{i}}$ indicates whether post *i* is related to the HBP platforms. *X*_*it*_ is a vector of post and user-level characteristics including geographic and gender diversity, posts including code snippets, and HBP partnership affiliation; we use shares instead of levels to account for the standardized user composition. *δ*_*t*_ denotes year-fixed effects. Heteroskedasticity-robust standard errors are reported. Given that most post-level interactions happen within the first three months, we do not have within-post over-time variation to allow for post-level fixed effects. All variables are aggregated to the post-quarter level.

Table 2 reports the results from post-level analyses. Column (1) includes covariates related to post composition that vary at post-level. Column (2) comprises initial post characteristics. Column (3) combines the two sets of covariates. Column (4) further includes topic category fixed effects to account for differences in the underlying post topics. Throughout the specifications in columns (1)-(3) user interactions are significantly higher for posts related to the HBP platforms, and programming posts with code snippets. Posts with a higher share of code-embedded replies have on average more replies. In particular, the inclusion of code snippets in the initial question post is a strong predictor of more follow-up interactions, with the estimates positive and statistically significant in all specifications. This is consistent with prior studies on the importance of including code snippets in the initial question to receive more attention of fellow users, and thus a faster and more targeted solution [33, 34].

**Table 2:** HBP Forum utilization analyses at the post-quarter level.

Dependent variable:	# replies per post-quarter			
	(1)	(2)	(3)	(4)
HBP platforms related	1.085***	1.843***	0.872***	
	(0.240)	(0.310)	(0.260)	
% replies w/ programming code	0.910***		0.635*	0.599*
	(0.336)		(0.340)	(0.348)
% female users replying	0.983		1.171	0.984
	(0.709)		(0.753)	(0.752)
% replying users w/ HBP affil.	0.579*		0.516	0.542*
	(0.315)		(0.327)	(0.328)
% senior users replying	0.374		0.424	0.459
	(0.349)		(0.340)	(0.350)
% admin users replying	-0.674**		-0.601*	-0.691**
	(0.334)		(0.339)	(0.343)
# countries	1.965***		2.056***	2.022***
	(0.288)		(0.333)	(0.326)
Initial post w/ programming code		0.766***	0.633**	0.600**
		(0.274)	(0.254)	(0.254)
Initial post by user w/ HBP affil.		-0.304	-0.0118	-0.0693
		(0.237)	(0.235)	(0.235)
Initial post by female		0.350	-0.134	-0.187
		(0.326)	(0.301)	(0.308)
Initial post by senior		-0.323	-0.0931	-0.0552
		(0.414)	(0.392)	(0.398)
Category Brain Sim/Model				-1.841
				(1.163)
Category Neurorobotics				-0.163
				(1.075)
Category Tech Support				-0.278
				(1.102)
Category Organization				-1.161
				(1.086)
Category Others				-1.190
				(1.080)
LHS mean	3.86	3.86	3.86	3.86
Year Fixed Effects	YES	YES	YES	YES
Observations	599	599	599	599

Notes: Each cell reports the coefficient of interest from a separate regression. Each unit is aggregated to the post-quarter level. We capture post-quarter user interaction by regressing the number of replies a post received on various covariates. Robust standard errors reported in parentheses.

^a^ Robust p-values:

*** p<0.01,

** p<0.05,

* p<0.1.

Contrary to prior studies [35, 36], we do not find statistically significant differences in user interaction patterns related to the gender of the user who raises a question. Similarly, whether a question is asked by a user with advanced access to the HBP platform or by a more senior user does not significantly alter the interaction on the Forum. These results likely partly reflect the usage of gender-neutral usernames and the lack of a direct information tag on user’s HBP affiliation status or experience on their profile and posts. This design feature of the Forum is worth further investigation in future studies of online institution building. In contrast, Forum administrators are clearly marked on the profiles and a higher share of replies from administrators (i.e., more institutional support provided) associates with lower intensity of voluntary interactions. Further, greater geographic diversity in participating users is associated with significantly more replies to a post within a quarter, and this effect is stronger when controlling for both the post-level and the initial post characteristics in column (3).

To account for differences between the underlying content discussed in each post, we further control for fixed effects at the content category level (column (4)). In this more demanding specification, the $HBP_{platform_{i}}$ variable is no longer present due to collinearity with the category fixed effects. After controlling for this content-level measure, more diverse country-level user participation (i.e., at the extensive margin) remains statistically significant and positively associated with active user interactions (i.e., at the intensive margin). Overall, we observe similar patterns in the main estimates. There is a higher level of user interactions for posts asking questions related to the HBP platforms, those including code snippets in the initial posts and in the replies, and those with geographically-diverse users.

### 3.2 What factors make problem solving faster on the HBP Forum?

**Fig 3:**
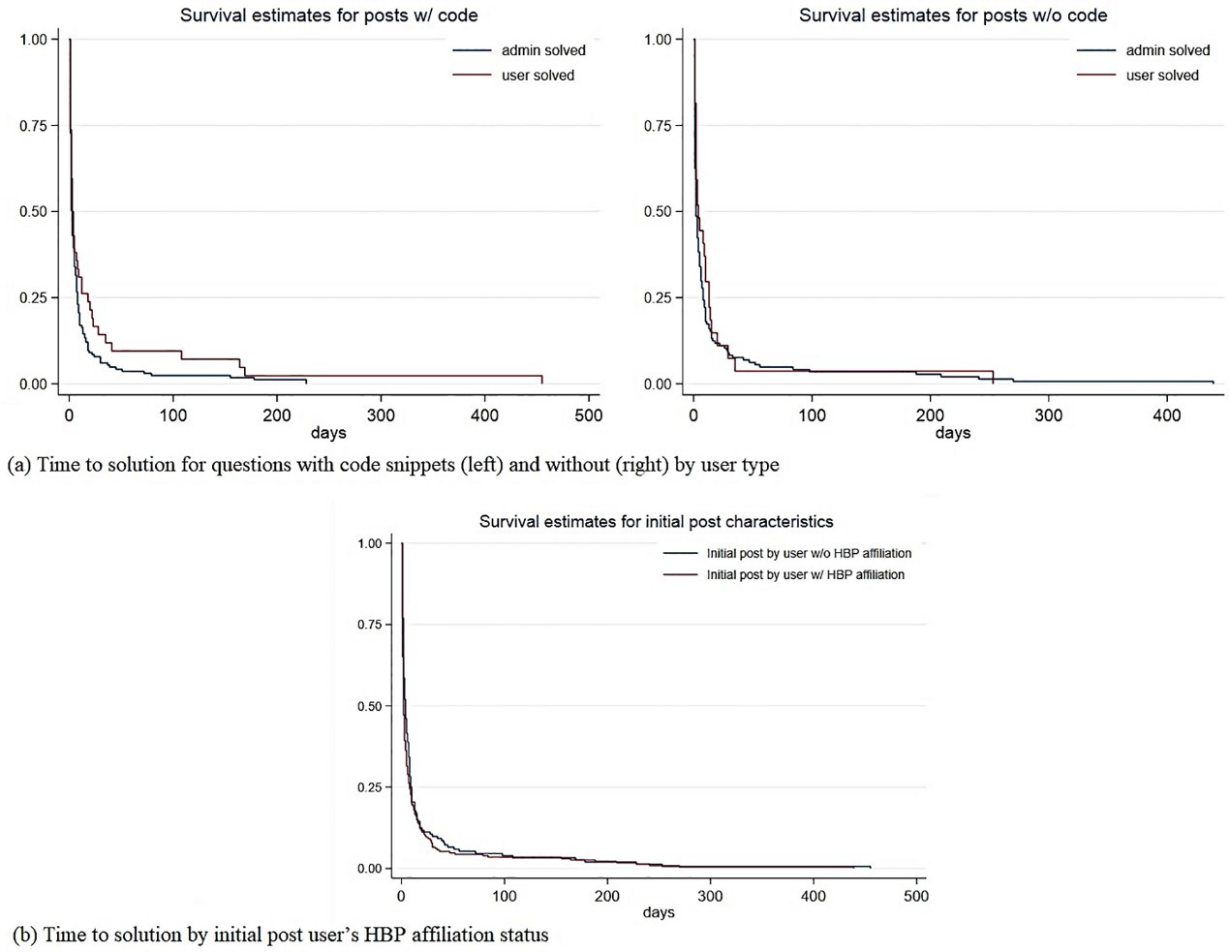
Kaplan-Meier non-parametric survival analyses. Notes: This figure reports a set of main graphical results from the survival analyses to disentangle what factors are most relevant for effective problem solving in online platforms. Additional graphs on other factors in the hazard and time-to-event analyses are available upon request.

To further examine the factors that accelerate research problem solving in the HBP Forum, we start with Kaplan-Meier non-parametric survival estimates to visually represent differences in problem solving associated with a few key factors. The Kaplan-Meier survival estimates represent the probability of an event occurring after a certain point in time. Fig 3(a) suggests that HBP Forum administrators solve programming-related questions faster than users. However, the difference in problem solving time for non-programming related topics is minimal between administrators and users. The sharp drop in the share of unsolved posts to 25% within the first 25 days suggests that 75% of questions posted, programming-related or not, are solved by then. Fig 3(b) suggests that solving time does not differ much between posts initiated by users affiliated with an HBP partner institution or not, which reflects our results regarding the usage analysis in Section 3.1.

To systematically understand factors associated with online problem solving, we use a Cox proportional hazard model to analyze solving time (in days) of the question raised in post *i*. For the general model, we apply the following functional form:

$$h\left(d_{i},\;s_{i},\;X_{i}\right)=h_{0}\left(t\right)exp^{\lambda_{i}\left(\alpha d_{i}+\beta X_{i}\right)}$$ (2)

where *h*_0_(*t*) indicates the baseline hazard. *s*_*i*_ indicates whether the questions raised in post *i* is solved by the end of our sample period. *d*_*i*_ is the number of days between when question *i* is raised and solved on the HBP Forum. *X*_*i*_ contains an indicator of the relevance of a topic to the HBP platforms, as well as the set of time-invariant covariates about the post-level characteristics that we used in the previous analyses (section 3.1). The coefficient estimates represent hazard ratios, and a ratio greater (smaller) than one indicates a positive (negative) relationship with the probability of a post being solved (i.e., with *s*_*i*_ equals one). As a robustness check, we also conducted a time-to-event analysis that allows for a more flexible functional form of the baseline hazard by transforming the survival function using natural cubic splines as a link function [37].

Columns (1)-(4) of Table 3 report the results of the Cox proportional hazard model. Column (1) includes the post profile covariates, column (2) the initial post characteristics, and column (3) a combination of both. Column (4) replaces the capture-all HBP Forum indicator with content-based topic category fixed effects. Column (5) shows the results of the time-to-event analysis using the same covariates as in Column (3). The results in columns (1)-(3) suggest that posts related to HBP platforms have a higher probability of being solved compared to non-platform related posts. The coefficient magnitudes are similar across specifications, and indicate that solving probability is on average twice as high for platform-related posts (e.g., Column (3): 1.956–1 = 0.956). The estimate is similar and larger in magnitude in the time-to-event analysis.

**Table 3:** Cox proportional hazard analyses and time-to-event analysis.

	Cox regression hazard ratios				Time-to-event hazard ratios
	(1)	(2)	(3)	(4)	(5)
HBP platforms related	1.975**	1.837*	1.956**		2.168*
	(0.617)	(0.590)	(0.618)		(0.862)
Solved by admin	1.293*	1.283*	1.302*	1.331**	1.366**
	(0.177)	(0.165)	(0.180)	(0.186)	(0.199)
% replies w/ programming code	1.339**		1.265*	1.264*	1.398*
	(0.160)		(0.153)	(0.156)	(0.246)
% female users	0.949		0.830	0.928	0.868
	(0.335)		(0.359)	(0.422)	(0.506)
% users w/ HBP affil.	1.331		1.348	1.249	1.386
	(0.238)		(0.292)	(0.279)	(0.346)
% senior users per post	1.153		1.153	1.218	1.227
	(0.174)		(0.187)	(0.201)	(0.226)
# countries	0.792***		0.794**	0.768***	0.737***
	(0.0696)		(0.0720)	(0.0744)	(0.0666)
Initial post w/ programming code		1.191*	1.102	1.128	1.127
		(0.116)	(0.111)	(0.114)	(0.133)
Initial post by user w/ HBP affil.		1.127	0.973	0.979	0.970
		(0.105)	(0.112)	(0.113)	(0.129)
Initial post by female		1.125	1.110	1.169	1.087
		(0.149)	(0.189)	(0.214)	(0.235)
Initial post by senior		1.080	0.990	1.105	1.003
		(0.182)	(0.164)	(0.171)	(0.182)
Category Brain Sim/Model				2.238**	
				(0.851)	
Category Neurorobotics				1.922**	
				(0.574)	
Category Tech Support				1.519	
				(0.502)	
Category Organization				1.331	
				(0.523)	
Category Others				2.299*	
				(1.089)	
Year Fixed Effects	YES	YES	YES	YES	YES
Observations	381	381	381	381	381

Notes: We report the results from estimating a Cox proportional hazard model and a time-to-event analysis for the time in days to a first solution of the post. We report hazard ratios. Hazard ratios greater (smaller) than one indicate a positive (negative) relationship with the probability of solving the post. The percentages reported are obtained by subtracting 1 from the hazard ratio coefficients. Robust standard errors are reported in parentheses.

^a^ Robust p-values:

*** p<0.01,

** p<0.05,

* p<0.1.

When administrators participate, the probability that a question posted on the Forum is solved is 28–33% (e.g., Column (3) 1.302–1 = 0.302) higher in the proportional Cox hazard model, and 37% higher in the time-to-event analysis. Combined with previous results that higher level of administrator participation associates with lower voluntary user interaction, our findings suggest that support from administrators solve questions effectively faster. In contrast to the wholly user-driven Stack Overflow experience [33], institutional support by the administrators appears to be very beneficial for Forum users. Questions on programming issues with code and related to HBP platforms are also solved faster. In column (4), the estimates on the categories “Brain Simulation and Modelling” and “Neurorobotics” re-enforce the results that platform-related posts and application questions are solved faster.

Consistent with our usage analysis, neither the gender of the user raising the question nor that of those who reply is statistical-significantly associated with lower solving time. We find no evidence for the differences in online knowledge sharing between women and men as observed in other studies [38]. The solving probability is also not affected if a higher share of senior researchers participate.

In the previous analysis, larger geographical user diversity is associated with richer user interaction. The results in Table 3, however, suggest a 21–23% lower probability of the post being solved (e.g., Column (3): 0.794–1 = -0.206) as the number of user-participating countries increase. The results are similar using the Cox proportional hazard model and time-to-event analysis. This result could imply that more time is needed in the accumulation and harmonization of knowledge from various sources, and that questions attracting more geographically diverse user participation may be more complex. In the absence of a clear metric for question complexity, we leave a fuller investigation of this for future research.

### 3.3 Additional discussions

In addition to providing an online community for neuroscientists, the HBP Forum also promotes open-source programming for back-end activities. While the HBP Forum provides direct user support on all HBP-related topics, some questions about the neurorobotics platform require further technical support and may be forwarded to an administrator-only repository–the neurorobotics Jira BitBucket, where more substantial issues and bugs are tracked and resolved. This repository is maintained by 14 out of the 50 HBP Forum administrators in our sample. In our sample, 18 out of 534 posts were sent to Jira BitBucket. In a robustness test, we control for whether a post was forwarded to BitBucket in the regressions to proxy for complexity. Our results remain unchanged, as the set of questions forwarded outside the Forum is small. In addition, most user interactions are voluntary instead of directed communications between users with prior ties: in only 25 posts we observe direct user tagging for additional support, and only 14 users were ever tagged directly (among which, nine were administrators).

Our result on female participation is in line with results of gender studies in online platform collaboration. Female users are in general under-represented in online technology-related Q&A platforms [38]. Studies show that female users are more inclined to participate in posts if there are already other female users replying to a question [35, 36]. Similar to Stack Overflow, the gender and affiliation of active users are not revealed in the user profile and as such, users are left to guess gender [35, 38]. This gender-neutral feature of platform design is worth further investigation and can potentially reduce gender-related biases.

## 4. Conclusion

We study the utilization and effectiveness of the HBP Forum, a platform that offers neuroscience researchers a public space to discuss issues and raise queries broadly related to the Human Brain Project. We construct a novel and comprehensive dataset to capture the usage of the HBP Forum, including the range of topics being discussed, plus whether, how quickly, and by whom research problems are solved. We find that the HBP Forum is actively used and remains active during Covid-19. On average, each forum post is discussed at a similar interaction level as those on general platforms (e.g., Stack Overflow), and Forum usage recovered fairly quickly after an initial drop of activity during Covid-19 first wave.

Forum posts generate more discussions on topics related to the HBP platforms, on questions related to programming, and when the topic attracts more interest from users based in different countries. Most questions posted on the HBP Forum are solved within 16 days, and questions are solved faster when forum administrators participate and when code snippets are included. The Forum appears to be an inclusive online community, where the usage and discussions do not significantly differ across HBP affiliation. We find no evidence that the gender or seniority level of users alter the discussion intensity or problem-solving probability.

Our results provide encouraging evidence that the online community built through the HBP has generated active participation among users from different institutions and with different educational levels who may not have otherwise connected. The institutional support provided by forum administrators appears helpful in supporting the collaborative progress of the online neuroscience community, which may be especially important at the current time, when physical distance to peers is increased. Our analyses offer a first glimpse into the facets of a particular online collaboration infrastructure within a large, long-term life science project.

## Supporting information

S1 Fig HBP Forum usage: Topics by content category. Notes: This histogram depicts the proportion of topic categories discussed on the HBP Forum overtime in quarterly units. The major content categories are created based on the official category tags generated on the HBP Forum. We grouped similar tags into major groups in line with the main subproject areas of the HBP. Among the six content categories, Neurorobotics, Neuromorphic, and Brain Simulation/Model are categories closely tied with HBP platform-based subproject areas.
(TIF)

S2 Fig Snapshot example of an answer to questions on neurorobotics (with light code). Notes: This figure provides an example of a question with light code discussed on the HBP Forum.
(TIF)

S3 Fig Snapshot example of a question on neurorobotics (with a block of code). Notes: This figure provides an example of a question with a block of code discussed on the HBP Forum.
(TIF)

S4 Fig Snapshot example of an organizational issue discussed (without code). Notes: This figure provides an example of a question without code discussed on the HBP Forum. More examples on the typical interactions between users within each content categories are available upon request.
(TIF)
