# Supplementary figures and images for: Collaborating neuroscience online: The case of the Human Brain Project forum
Source: PLoS One. 2022 Dec 7;17(12):e0278402. doi: 10.1371/journal.pone.0278402 (PMC9728874; doi:10.1371/journal.pone.0278402)

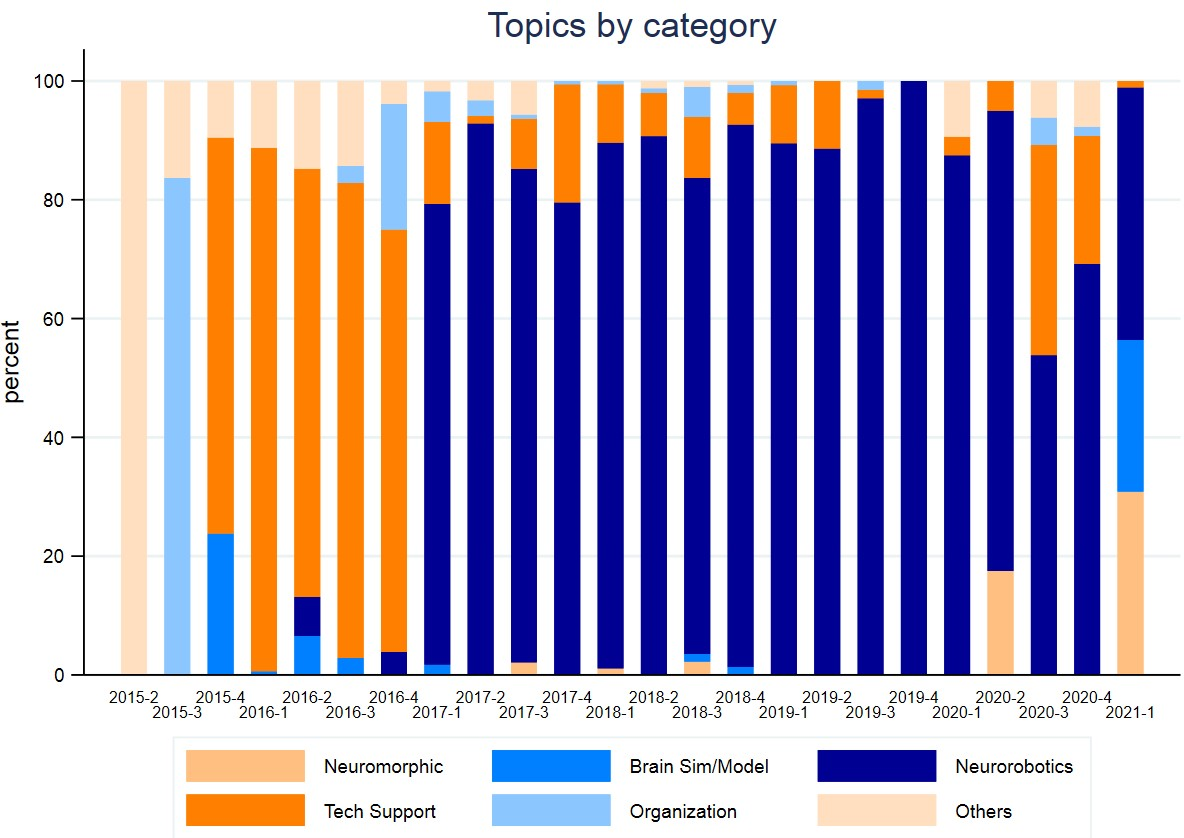

Supplement: S1 Fig — Notes: This histogram depicts the proportion of topic categories discussed on the HBP Forum overtime in quarterly units. The major content categories are created based on the official category tags generated on the HBP Forum. We grouped similar tags into major groups in line with the main subproject areas of the HBP. Among the six content categories, Neurorobotics, Neuromorphic, and Brain Simulation/Model are categories closely tied with HBP platform-based subproject areas. (TIF) [file pone.0278402.s001.tif]

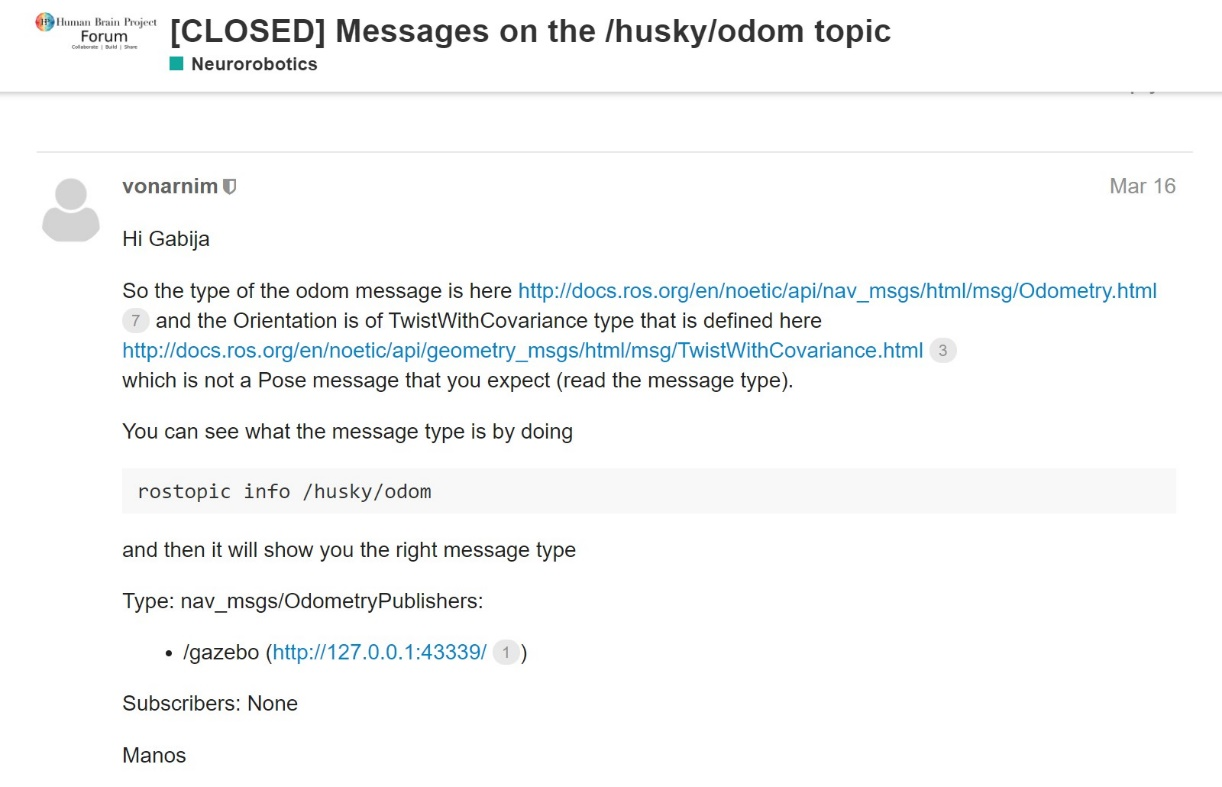

Supplement: S2 Fig — Notes: This figure provides an example of a question with light code discussed on the HBP Forum. (TIF) [file pone.0278402.s002.tif]

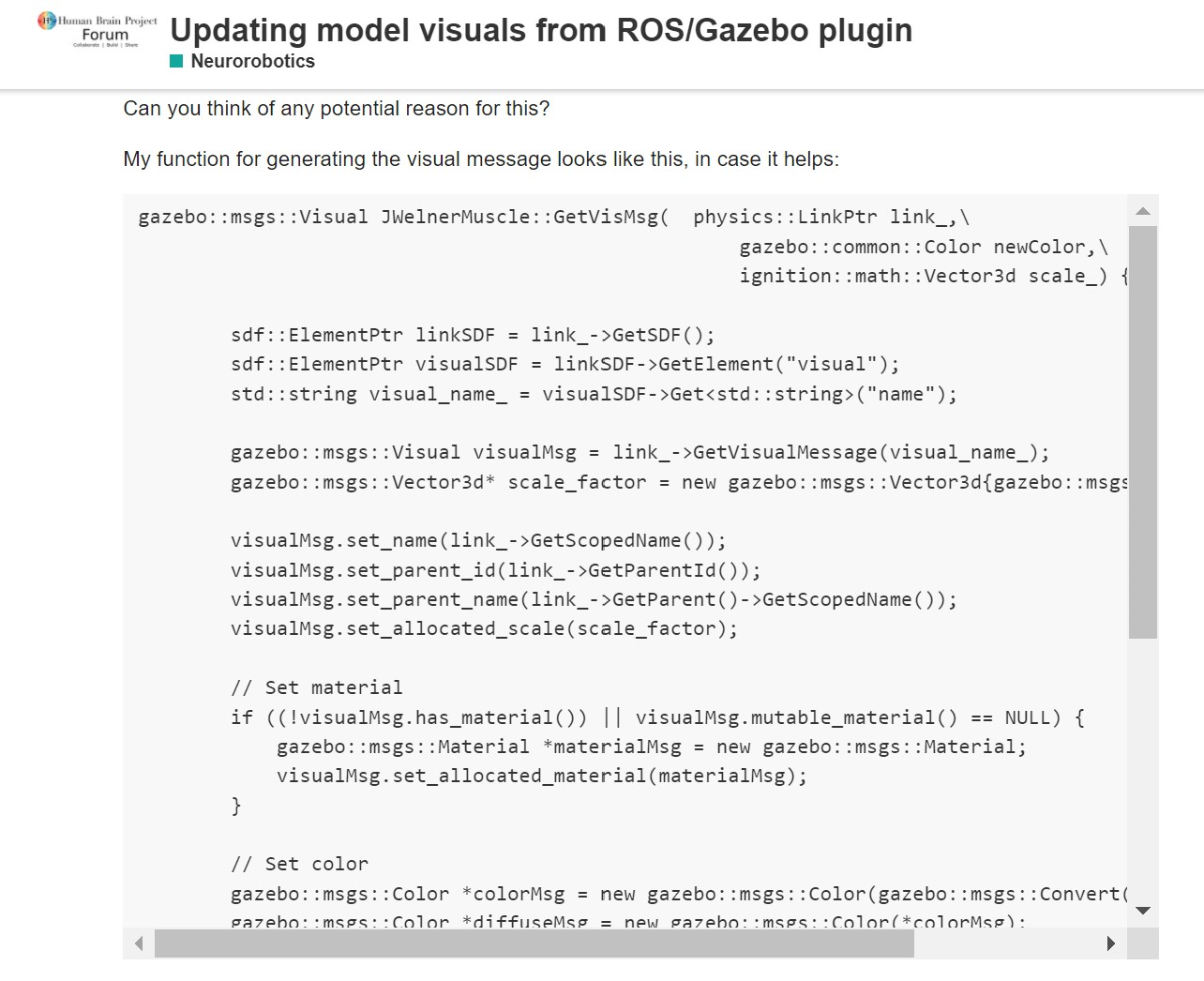

Supplement: S3 Fig — Notes: This figure provides an example of a question with a block of code discussed on the HBP Forum. (TIF) [file pone.0278402.s003.tif]

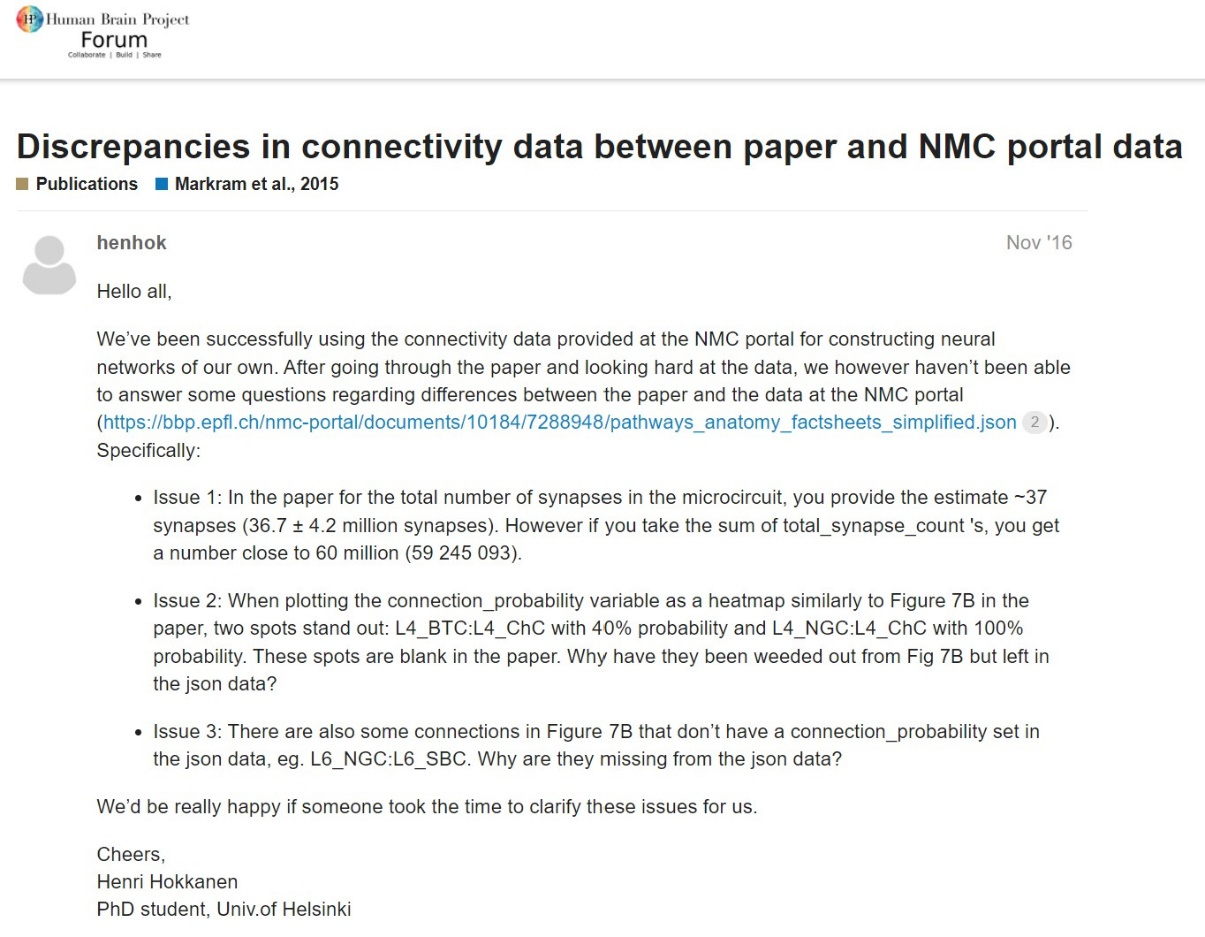

Supplement: S4 Fig — Notes: This figure provides an example of a question without code discussed on the HBP Forum. More examples on the typical interactions between users within each content categories are available upon request. (TIF) [file pone.0278402.s004.tif]
